# Inducing fear using acoustic stimuli—A behavioral experiment on moose (*Alces alces*) in Sweden

**DOI:** 10.1002/ece3.9492

**Published:** 2022-11-16

**Authors:** Manisha Bhardwaj, Denice Lodnert, Mattias Olsson, Aina Winsvold, Svein Morten Eilertsen, Petter Kjellander, Andreas Seiler

**Affiliations:** ^1^ Department of Ecology Swedish University of Agricultural Sciences Riddarhyttan Sweden; ^2^ Faculty of Environment and Natural Resources, Chair of Wildlife Ecology and Management University of Freiburg Freiburg Germany; ^3^ EnviroPlanning AB Gothenburg Sweden; ^4^ Ruralis – Institute for Rural and Regional Research University Centre Dragvoll Trondheim Norway; ^5^ Norwegian Institute of Bioeconomy Research Ås Norway

**Keywords:** acoustic deterrent, anti‐predatory behavior, human–wildlife interactions, hunting pressure, landscape of fear, predation, wildlife management

## Abstract

Prey species may display anti‐predatory behavior, i.e., flight, increased vigilance, and decreased feeding, in response to the true presence of a predator or to the implied presence of a predator through, e.g., acoustic cues. In this study, we investigated the anti‐predatory reactions of moose (*Alces alces*) to acoustic stimuli related to hunting, at saltlick stones, a known attractant. In before‐during‐after‐control‐impact experiments, we compared the behavioral responses of individuals to: (i) two hunting‐related acoustic stimuli—hunting dog barking and human speaking; (ii) nonpredatory acoustic stimuli—bird sounds and; and (iii) no acoustic stimulus (control). We asked: (1) How does the probability of moose leaving the site differ depending on the stimulus they are exposed to?; (2) What affect do the acoustic stimuli have on the amount of time moose spend vigilant, feeding, or away from the site?; and (3) What affect do the stimuli have on the time between events at a site? We found that when exposed to the human stimulus, moose left the sites in 75% of the events, which was significantly more often compared to the dog (39%), bird (24%), or silent (11%) events. If moose did not leave the site, they spent more time vigilant, and less time feeding, particularly when exposed to a dog or human stimulus. Furthermore, moose spent the most time away from the site and took the longest to visit the site again after a human stimulus. Moose were also more likely to leave the site when exposed to the bird stimulus than during silent controls. Those that remained spent more time vigilant, but their behaviors returned to baseline after the bird stimulus ended. These findings suggest that acoustic stimuli can be used to modify the behavior of moose; however, reactions towards presumably threatening and nonthreatening stimuli were not as distinct as we had expected.

## Graphical abstract

When moose are exposed to human voices, they have a high likelihood to leave the given site and this relationship remains high after 50 days of exposure to the sound.

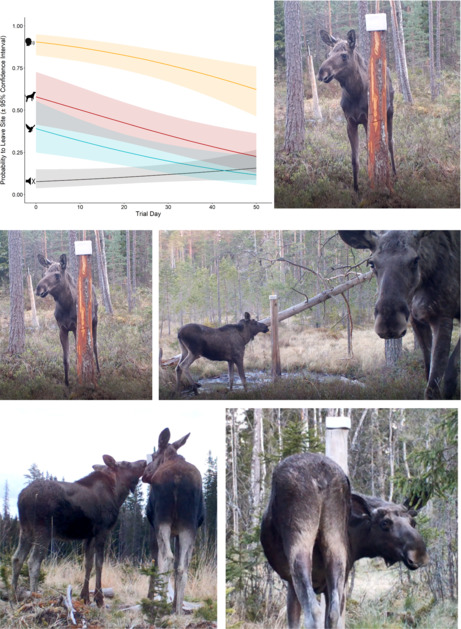

## 1 INTRODUCTION

Fear in animals is the degree of risk or threat animals perceive in a given situation (Stankowich & Blumstein, 2005) and can motivate a change in the individual's behavior (Brown et al., 2012; Stankowich & Blumstein, 2005). Behavioral responses as a result of fear are often innate and formed through evolution (Sih et al., 2004). Some behaviors commonly occur together. For example, ungulates have a suite of “anti‐predatory” behaviors, which include flight, increased vigilance, and decreased feeding (Brown et al., 2012; Brown & Kotler, 2004; Frid & Dill, 2002; Kuijper et al., 2014; Stankowich, 2008; Stankowich & Blumstein, 2005). The behavior demonstrated is a result of the trade‐off among energy expenditure, resource allocation, and individual safety, which in turn has an influence on the success of the individual and population (Creel & Christianson, 2008).

Anti‐predatory responses in ungulates can be motivated by the presence of a threat, such as predators (e.g., wolves, *Canis lupus*) or humans, in their environment (Brown et al., 2012; Stankowich & Blumstein, 2005). However, these behaviors can also be artificially induced in the absence of a predator. For example, flight or increased vigilance in ungulates can be achieved through visual stimuli, such as lights and moving objects (Koehler et al., 1990); olfactory stimuli, such as wolf urine (Chamaillé‐Jammes et al., 2014); and acoustic stimuli, such as the sound of a predator or the alarm calls of conspecifics (Babińska‐Werka et al., 2015). Of these methods, acoustic stimuli tend to be the most effective at inducing fear in ungulates, as, when they are naturally occurring, these cues indicate spatial and temporal proximity of a threat that prey respond consistently towards over time (Biedenweg et al., 2011; D'Angelo, 2007; Hettena et al., 2014; Lutz, 1994; Seiler et al., 2017; VerCauteren et al., 2003).

Anti‐predatory behavioral displays in moose are strongly dependent on the experience of moose to predatory threats and the environment they inhabit. In Scandinavia, moose tend to respond strongly to human‐recreational activity in their environment (e.g., hunting, hiking, snowmobiling; Neumann, 2009), while the presence of wolves has little influence (Månsson et al., 2007; Nicholson et al., 2014; Sand et al., 2006, 2021; Wikenros et al., 2016). This may be due to the fact that, until the 1980s, wolves were extirpated from this region (Wabakken et al., 2001), and hunting has been the primary source of mortality in moose, even while moose are within wolf territories (Lavsund & Sandegren, 1989; Stubsjoen et al., 2000; Wikenros et al., 2016; Zimmermann et al., 2019). While most studies focus on behaviors such as habitat selection, movement trajectories, and space use of moose in the true presence of threat (Neumann, 2009; Nicholson et al., 2014; Sand et al., 2006, 2021; Wikenros et al., 2016), few studies in Scandinavia have evaluated the response of individual moose to simulated predation threat (for exceptions, see Berger, 2007; Berger et al., 2001).

In this study, we aimed to evaluate whether moose display behavioral changes when exposed to acoustic stimuli and whether there is a difference in response depending on the type of acoustic stimuli used. To achieve this, we compared how wild living moose in Sweden responded to acoustic stimuli while visiting saltlick stones. Given what is known about the display of anti‐predatory behavior in Scandinavian moose, we chose to use human‐related cues rather than wild‐predator‐related cues. Therefore, we compared the responses of moose towards threatening hunting‐related stimuli: dog barking and human voice, with nonthreatening stimuli: bird sounds, and with no acoustic stimulus displayed (i.e., silent controls). The anti‐predatory behaviors we were interested in were: increased flight, increased vigilance, reduced feeding, and site avoidance. We asked: (1) How does the probability of moose leaving the site differ depending on the stimulus they are exposed to?; (2) What affect do the acoustic stimuli have on the amount of time moose spend vigilant, feeding, or away from the site?; and (3) What affect do the stimuli have on the time between events at a site? We predicted that moose will display anti‐predatory behaviors, i.e., flight and vigilance, significantly more when exposed to threatening stimuli than nonthreatening stimuli or to the “normal” situation, i.e., during the silent controls. Furthermore, we predicted that there would be more time in between events where moose were exposed to threatening stimuli than when exposed to nonthreatening stimuli or in control situations.

## 2 METHODS

### 2.1 Study area

**FIGURE 1:**
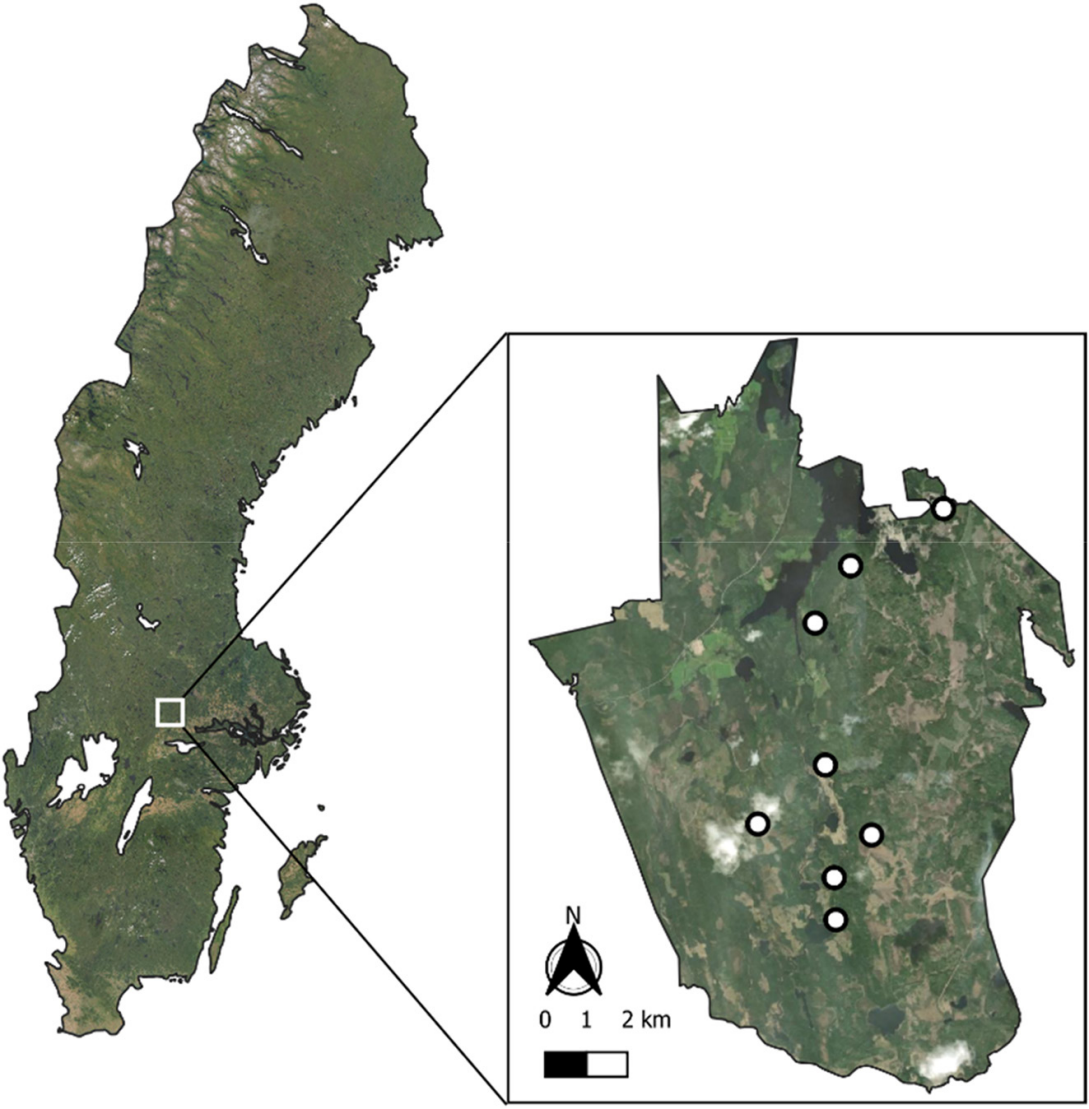
Location of the Grimsö Wildlife Research Area in Sweden and the 8 sites (white circles) within the research area where the experiments were conducted (*source*: Esri, 2020).

We conducted this study in the Grimsö Wildlife Research Area in south‐central Sweden (59.7286 N, 15.4724 E; Figure 1). Moose are a common, widespread species in Sweden (Bergqvist et al., 2002), and in the research area, the density of moose is 11 moose/1000 ha (ÄSO, 2020). The research area comprises 13,000 ha, dominated by forest consisting of Scots pine (*Pinus sylvestris*) and Norway spruce (*Picea abies*) and 18% of the research area consists of boggy wetlands (Faber, 1998; Månsson et al., 2007). The forest is owned by the state and managed by Sweden's largest forest company *Sveaskog*, a profit‐driven forestry company. Stands of spruce and pine forests are regularly clear cut in rotation (60–120 years) throughout the research area. The research area is also a common area of human recreation. Within the research area ungulates (moose, wild boar [*Sus scrofa*], roe deer [*Capreolus capreolus*] and red deer [*Cervus elaphus*]), other mammals (e.g., red fox [*Vulpes vulpes*], European hare [*Lepus europaeus*], Eurasian beaver [*Castor fiber*]), and fowl are hunted for population control purposes. The annual hunting season begins in August and ends in March depending on the species; for moose, the hunting season is from October to January. Dogs are commonly used when hunting.

### 2.2 Experimental design

Since 1972, saltlick stones have been used in the Grimsö Wildlife Research Area to attract moose, particularly in spring and early summer when moose seek resources to increase their sodium intake (Laurian et al., 2008). We collected data at 8 of these pre‐established saltlick stone sites, positioned at least 1 km apart (Figure 1). Preliminary monitoring of these sites showed that moose tend to spend 2 min or fewer at the saltlick stones per visit.

To evaluate the response of moose to different acoustic stimuli, we used the “Motion‐Activated Scaring System” (MASS), a system comprising a computer that displays an acoustic stimulus when activated by motion. The system was connected to a *Reconyx Hyperfire 2* wildlife camera (Reconyx Inc., 2015), which recorded 60‐s videos when the MASS was activated. The videos consisted of three parts: 20 s of silence *before* an acoustic stimulus was displayed, 20 s *during* the display of an acoustic stimulus, and 20 s *after* the stimulus was displayed. The MASS system and the camera were placed 10 m from the saltlick stone at each site to allow for the widest frame of view while remaining within the limits for the sensor to be activated by movement. The MASS was modeled after a similar system created by Suraci et al., 2017.

### 2.3 Acoustic stimuli

We aimed to study if moose would respond differently depending on the context of the stimuli used. To address this, we used presumably threatening stimuli: a dog barking and a human voice, and presumably nonthreatening stimuli: bird sounds common in the research area (Hettena et al., 2014). The threatening stimuli were those associated with hunting; the barking dog was recorded from a dog used for moose hunting and the human voice was a male talking in a normal conversational tone to simulate the presence of a person in the forest. The nonthreatening stimuli were the song of a Boreal Owl (*Aegolius funereus*), and the drumming of a Black woodpecker (*Dryocopus martius*) (Swedish Bird Altas, 2021). The Boreal Owl song was used during night (22:00–03:59), and woodpecker drumming was used during the daytime to represent when that species is active. The selection of threatening and nonthreatening acoustic stimuli is similar to those in other playback experiments (e.g., Clinchy et al., 2016; Crawford et al., 2022; Epperly et al., 2021; Smith et al., 2017; Suraci et al., 2019; Widén et al., 2022). Each acoustic stimulus was contained on a single audio file (i.e., a single exemplar of each stimulus).

When the MASS was triggered, one of the four stimuli would randomly display after a silent period of 20 s, as previously described. Once triggered, the MASS could not be triggered for another 3 min, to reduce the amount of exposure to acoustic stimuli, and to avoid the risk of creating overly‐disturbed areas where the moose stopped visiting entirely. Every second trigger was silent to act as a control, in order to allow us to observe moose behavior while they were undisturbed and to detect if there are any behavioral changes due to electronic sounds emitted from the MASS units, which could not be detected with the human ear.

### 2.4 Data collection

We conducted the experiment from 21 May 2020 to 9 July 2020 (50 days of data collection), while moose were most active, i.e., 18:00–9:59. Over the remaining hours of the day, the MASS was set to only display the silent control. This was to avoid a large number of false activations by, for example, birds or moving vegetation. We visited each site every third day to collect memory cards and change batteries. At each visit, we used the “*Decibel X*” (SkyPaw Co Ltd., www.skypaw.com/decibelx.html) app on an Apple iPhone 7 to collect decibel levels before and after changing batteries in order to ensure that the loudness of the stimuli remained at the same level while battery levels decreased. Throughout the experiment, the decibel level ranged between 60–70 dB for all three acoustic stimuli when standing 1 m from the speakers.

Meteorological parameters, rain and wind, can cause refraction, scattering, and absorption of sound waves, which can influence sound propagation (Trikootam & Hornikx, 2019; Ziemann et al., 2016). They have also been shown to cause changes in ungulate behavior and occurrence (Herfindal et al., 2019). To account for these impacts, we recorded the amount of rain and wind speed at the site during the hour of the events (LantMet, 2020).

### 2.5 Behavior analysis

We quantified individual behavior from the videos using the open‐access software “*BORIS*” (https://www.boris.unito.it/; Friard & Gamba, 2016). We recorded how long each individual captured on video spent performing common behaviors, such as feeding, vigilance, and flight (Table 1). Time spent on each behavior was rounded to the nearest second, and separated into the three 20‐s periods: “before”, “during” and “after” the acoustic stimulus (or silent stimulus) was displayed. (See Table S1 and Figure S1 for details on time spent displaying each behavior).

**TABLE 1:** Ethogram of the behaviors quantified in BORIS for each individual moose in the video.

Behavior	Description
Fleeing	Moose moved in a fast pace, seemingly disturbed by something
Vigilant	Moose displayed clear alert behavior (ears up and looking in a few directions), and were observant to the surrounding. If feeding, the moose stopped completely.
Feeding	Moose browsing on vegetation in the surrounding area around the saltlick stone or licking on the saltlick stone
Out of frame	Moose left the site or was no longer visible in the frame
Standing	Moose stood by the saltlick stone or in the surrounding, seemingly undisturbed
Social interaction	Moose displayed social interaction with another moose, either cooperative or competitive
Walking	Moose walked in a slow pace, seemingly not stressed or disturbed

From the video analysis, we focused on the relative amount of time each individual spent vigilant, feeding, or out of the frame in the given event. Since we could only quantify the amount of time an individual displayed a given behavior while they were visible on camera, we calculated the proportion of time according to the time the individual was visible. Each event was separated into three periods before, during, and after the acoustic stimulus. The time spent out of frame was used for two reasons: (1) from the silent controls, or the before periods, it was used to indicate how individuals would naturally leave and come back into the frame of the videos; and (2) during or after the acoustic stimulus, it was used as one measure of avoidance. We also accounted for whether or not the individual left the site, and how long it took for another event to occur at the same saltlick stone, in order to calculate the proportion of events in which the individual flees in response to the stimulus, and how long it takes moose to visit the site again, after the stimulus is displayed (Table 2). We did not have any marked individuals so it was not possible to know if each subsequent event involved the same individual or not. For each event, we also recorded the acoustic stimulus of the event and the event previous, the day of the trial on which the event occurred and the saltlick stone site that the data come from (Table 2).

**TABLE 2:** Response variables and explanatory variables used to analyze the behavioral response of moose to different acoustic stimuli.

Variable	Notation	Description
Response variables		
Leaving the site	*L*_i_	Binary variable quantifying if moose left the site during the video. Yes = 1, No = 0.
Vigilant	*B*_i_	The time moose spent vigilant proportional to the amount of time they were visible in the frame (range 0–1).
Feeding	*B*_i_	The time moose spent feeding proportional to the amount of time they were visible in the frame (range 0–1).
Time out of frame	*B*_i_	The time moose spent out of frame proportional to the total time (range 0–1).
Time between events	*R*_i_	Minutes elapsed since the last visit by moose at the same site.
Explanatory variables		
Stimulus	*s*_i_	Factor for each acoustic stimulus: dog, human, owl, woodpecker, or the silent control.
Trial day	*d*_i_	Trial day ranging from day 1 until day 50.
Period	*x*_i_	Factor with three levels: *before* exposure to acoustic stimulus or silent control (0:00–0:19 of each event), *during* exposure to acoustic stimulus or silent control (0:20–0:39 of each event), and *after* exposure to acoustic stimulus or silent control (0:40–0:59 of each event).
Age class	*j*_i_	Factor with two levels: adult or juvenile.
Sex class	*g*_i_	Factor with four levels: female, female with calf, male, or unknown (when sex was indeterminable).
Rain	*q*_i_	Amount of precipitation (mm/h) during the hour of the event.
Wind	*w*_i_	Wind speed (m/s) during the hour of the event.
Previous stimulus	*c*_i_	The stimulus played at the previous moose visit.
Random effects		
Site	*z*_i_	Factor of site ID 1–8.

*Note*: Notation refers to how the variable is represented in model specification. Vigilant, feeding, and time out of frame are all noted as “*B*_i_” since the same model specification was used to explore each response, even though each behavior was modeled separately.

Partway through the trials, females gave birth to calves; the first moose calves were observed in an event on 7 June 2020. To account for the potential differences between sexes and between age classes, we recorded whether the focal individual was male, female, female with a calf, and adult with indeterminable sex, or a juvenile (Table 2).

### 2.6 Statistical analysis

#### 2.6.1 How does the probability of moose leaving the site differ depending on the stimulus they are exposed to?

To explore changes in the probability to leave the site, we fitted a binomial regression model, using the variable leaving the site, *L*, as the response (Table 2). For each data point i (each event),

$$L_{i}∼\text{Binomial}\left(p_{i},n_{i}\right)$$

$$\begin{array}{c} \text{logit}\left(p_{i}\right)∼\alpha +\beta_{1}\left(s_{i}\right)\times \beta_{2}\left(d_{i}\right)+\beta_{3}\left(j_{i}\right)+\beta_{4}\left(g_{i}\right)+\beta_{5}\left(q_{i}\right)+\beta_{6}\left(w_{i}\right)+\varepsilon_{z\left(i\right)}, \end{array}$$

where *p*_i_ is the probability of an individual leaving the site. The stimulus used in the event is represented by *s*_i_. To detect signs of habituation towards a certain stimulus, we included an interaction term between stimulus and trial day (*d*_i_). To account for differences among age and sex classes, we included *j*_i_ and *g*_i_, respectively. Variations in environmental conditions were accounted for by including rain (*q*_i_) and wind (*w*_i_). Finally, we included a random‐effect term for site $\varepsilon_{z\left(i\right)}$ to account for local differences in site attraction and to also account for the fact that the same individuals are likely sampled multiple times (and more likely to repeatedly visit the same site), even though we cannot know for certain since individuals are not marked. *α* is the intercept, which represented adult females without calves during silent events on the first day of the experiments, with no rain and wind. Detailed explanation of each variable is available in Table 2. Since we were interested in exploring whether moose left more after being exposed to the given stimulus, moose that left the site within the first 20 s (i.e., the time before a stimulus) and did not return within the 60‐s video were excluded from these analyses (*n* = 104).

#### 2.6.2 What affect do the acoustic stimuli have on the amount of time moose spend vigilant, feeding, or away from the site?

To explore changes in behaviors of interest—vigilant, feeding, and away from the site (i.e., time out of frame)—as a result of the acoustic stimulus, we fitted three separate binomial regression models, using the proportion of time spent displaying the given behavior, *B*, as the response (Table 2). For each data point i (each event):

$$B_{i}∼\text{Binomial}\left(p_{i},n_{i}\right)$$

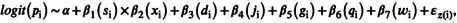

where *p*_i_ is the average proportion of time individuals spent performing the different behavior (*B*_i_). *s*_i_ represents the stimulus used in that event. To detect changes in the given behavior towards the stimulus throughout the event, we included an interaction term between stimulus and period (*x*_i_). To account for changes in responsiveness over the 50 days of the experiment, we included trial day (*d*_i_). Similar to the previous question, variations in age and sex were accounted for by including *j*_i_ and *g*_i_, respectively, environmental conditions were accounted for by including rain (*q*_i_) and wind (*w*_i_), and site‐level variation was accounted for by including a random‐effect term for site $\varepsilon_{z\left(i\right)}$. *α* is the intercept, which represented adult females without calves during the before the period of silent events on the first day of the experiments, with no rain and wind (Table 2).

#### 2.6.3 What affect do the stimuli have on the time between events at a site?

To explore how the stimuli affected the time between events at the same site, we fitted a gamma regression model using time between events, *R*, as the response (Table 2). For each data point i (each event):

$$R_{i}∼\text{Gamma}\left(\lambda_{i},v\right)$$

$$\begin{array}{c} \log\left(\lambda_{i}\right)∼\alpha +\beta_{1}\left(c_{i}\right)+\beta_{2}\left(d_{i}\right)+\beta_{3}\left(q_{i}\right)+\beta_{4}\left(w_{i}\right), \end{array}$$

where *λ*_i_ is the mean time until the next visit at the same site by a moose. *c*_i_ represents the stimulus used in the event previous to this one. We used the stimulus of the previous event to test whether the amount of time between events was related to exposure to a particular stimulus. As with the last two questions, we accounted for changes in responsiveness over the 50 days of the experiment by including trial day (*d*_i_), and variations in environmental conditions by including rain (*q*_i_) and wind (*w*_i_). In this analysis, site (*z*_i_) was removed due to issues of singularity, due to the fact that, on some trial days, moose visited a particular site once or not at all. Since we were interested in the change in responsiveness over time, we chose to keep the trial date rather than the site in the model. *α* is the intercept, which represented events in which moose were exposed to a silent event in the event previous, on the first day of the experiments, with no rain and wind. We do not have marked individuals at this site, and thus cannot tell which individuals are recorded in each event. As a result, we did not include age and sex into these analyses, since there was no way to tell if the individual was the same individual who received the previous stimulus. This means that the time between events cannot reflect the particular individual present in each event, however, gives a general estimate of the time between events.

We conducted all analyses in *R* (R Development Core Team, 2020), using the “glmer” function in the *lme4* package (Bates et al., 2015). We selected the most parsimonious models based on AIC corrected for small sample sizes (AICc; Akaike, 1973; Burnham & Anderson, 2002). We performed model selection on every model described above using the “dredge” function in the *MuMIN* package (Bartoń, 2013), and used the “mod.avg” function to average all top‐performing models (∆AICc < 2; Burnham & Anderson, 2002). The results we present are the conditional model averages. To compare the pairwise differences in moose responses to each acoustic stimulus, after each analysis we conducted post hoc Tukey's honest significant difference tests (Tukey, 1977) using the “glht” function in the *multcomp* package (Hothorn et al., 2008).

## 3 RESULTS

We collected a total of 4308 events, of which 701 were of moose (Table S2). The remaining events were of other wildlife such as European hare, roe deer, and birds. Five videos were too dark to analyze and were thus removed from the analysis. The final dataset analyzed consisted of 696 events, which displayed 761 individuals (Table 3).

**TABLE 3:** The number of events of moose exposed to the dog, human, bird, or silent control stimuli and the number of individuals in each period of the events.

	*N*_Events_	*N*_Individuals_		
		Before	During	After
Dog	142	158	138	110
Human	143	154	137	48
Bird	132	147	125	120
Silent	279	302	257	248
Total	696	761	657	526

*Note*: Changes in the number of individuals represent those moose leaving the site before the end of the event. Some events had more than one individual present.

There were 313 observations of males and 321 of females present in our trials (29 of the females had calves). In 127 events, it was not possible to tell if it was a male or female (41 of which were juveniles).

In our initial experimental setup, the owl song and woodpecker drumming were intended to represent the same type of stimulus—a nonthreatening, commonly heard sound. To test whether this was true, we compared the two stimuli to one another and determined there was no difference in the probability for moose to leave, display vigilance, feeding or time out of frame, or the time between events as a result of either stimulus (*n*_owl_ = 113; *n*_woodpecker_ = 34; probability to leave: *p* = .973; vigilance: *p* = .577; feeding: *p* = .185, time out of frame: *p*= .353; time between events: *p* = .915). As such, we combined the two stimuli into a single “bird” category for all subsequent analyses, and the stimulus variable was reduced to a factor of four categories: dog, human, bird, or silent.

### 3.1 Probability of leaving the site

**FIGURE 2:**
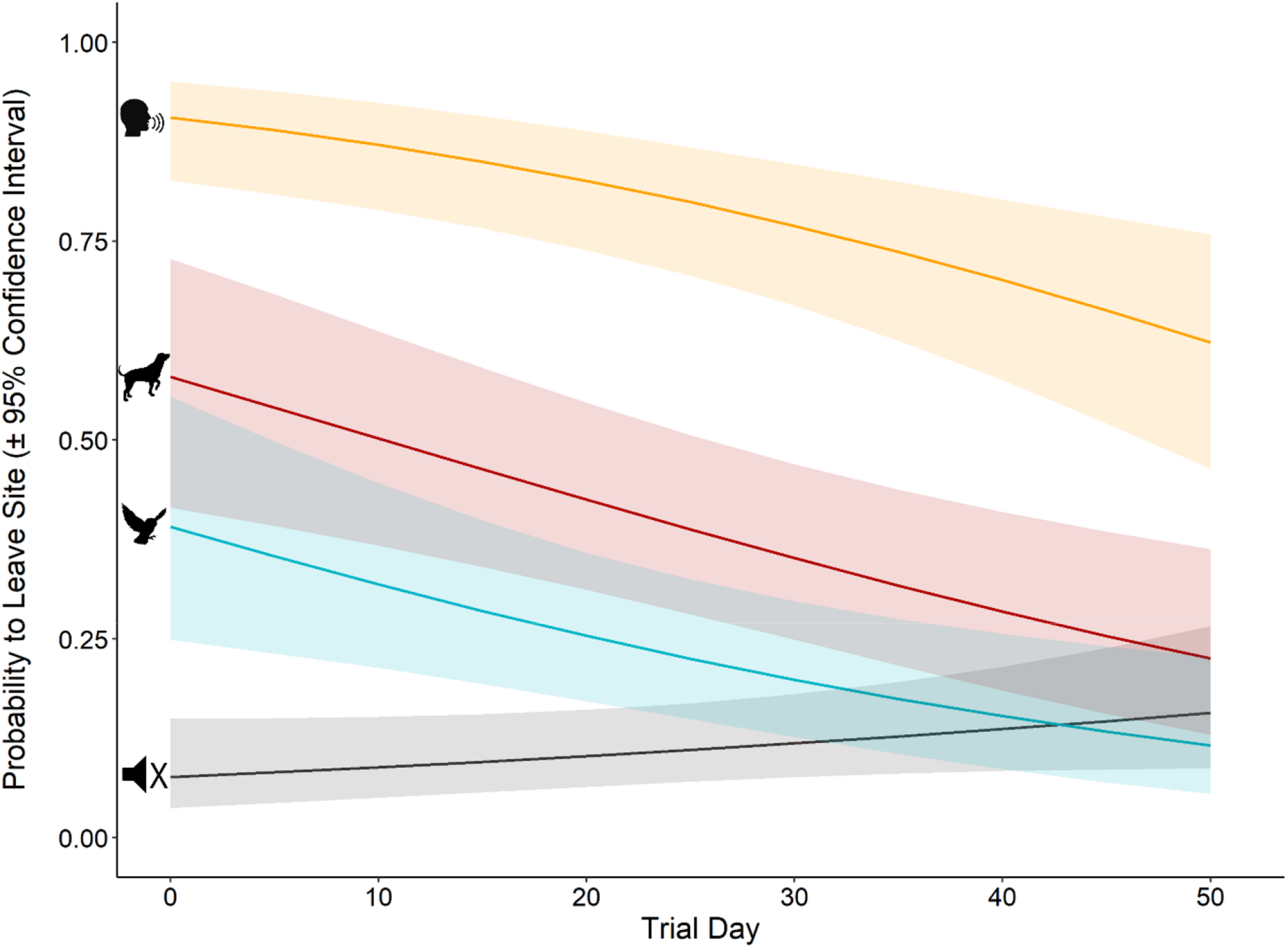
The estimated probability for moose leaving the site after being exposed to a dog, human, or bird stimulus, or the silent control, over the 50 days of the experiment. The lines reflect the model estimated probability to leave the site, and the shaded areas display 95% confidence interval for the given acoustic stimulus.

In the model describing the probability of a moose to leave the site, all variables present in the global model were also present in the top models (∆AICc < 2; Table 4) and were therefore included in the conditionally averaged logistic regression (Table 5). Moose had a higher probability to leave a site when exposed to any of the four acoustic stimuli, compared with the silent control (*n* = 29; *p* < .001; Figure 2). Moose exposed to human stimulus left more often (*n* = 103; 75% of events) compared with dog stimulus (*n* = 54; 39% of events) and bird stimulus (*n* = 30; 11% of events; Tables 5 and 6). Without accounting for a trial day, there was no significant difference in the overall probability for moose to leave after the dog stimulus and bird stimulus (*p* = .15; Table 6). Adult males were less likely to leave the site than adult females (*p* = .019); however, females with calves were most likely to leave the site (*p* = .003; Table 5). With increasing trial days, the probability for moose to leave a site decreased regardless of stimuli type (*p* ≤ .001), but there was no significant decrease in the probability to leave after exposure to the silent control (*p* = .11; Figure 2). Weather variables did not have a significant effect on the probability for moose to leave the site (rain: *p* = .64; wind: *p* = .83; Table 5).

**TABLE 4:** AICc table for the candidate models describing the probability for moose to leave the site after being exposed to an acoustic stimulus.

Candidate models	AICc	∆AICc	AICc weight
Intercept + Stimulus + Trial day + Stimulus × Trial day + Sex	1842.5	0	0.37
Intercept + Stimulus + Trial day + Stimulus × Trial day + Sex + Rain	1844.3	1.83	0.15
Intercept + Stimulus + Trial day + Stimulus × Trial day + Age + Sex	1844.3	1.83	0.148
Intercept + Stimulus + Trial day + Stimulus × Trial day + Sex + Wind	1844.5	1.98	0.137
Intercept + Stimulus + Trial day + Stimulus × Trial day + Age + Sex + Rain	1846.1	3.63	0.06
Null model	2405.2	562.73	0

*Note*: We display the top‐performing models (∆AICc < 2), the first model ∆AICc > 2, and the null model.

**TABLE 5:** Conditionally averaged model output for the probability that moose would leave a site after exposure to an acoustic stimulus.

Coefficients	Estimate	Standard error	*p* Value
Intercept	*−2.49*	*0.38*	*<.001*
Trial day	0.02	0.01	.113
Dog stimulus	*2.82*	*0.39*	*<.001*
Human stimulus	*4.76*	*0.42*	*<.001*
Bird stimulus	*2.04*	*0.40*	*<.001*
Dog stimulus × Trial day	*−0.05*	*0.01*	*<.001*
Human stimulus × Trial day	*−0.05*	*0.01*	*<.001*
Bird stimulus × Trial day	*−0.05*	*0.01*	*.001*
Juvenile	0.15	0.33	.659
Adult female with calf	*0.86*	*0.29*	*.003*
Adult male	*−0.33*	*0.14*	*.019*
Adult (unknown sex)	*0.52*	*0.20*	*.011*
Rain	−0.03	0.07	.638
Wind	0.01	0.06	.829

*Note*: The intercept is adult females (without calves) during silent events on the first day of the experiments, with no rain and wind. Variables with a significant effect (*α* = .05) are italicized.

**TABLE 6:** Pairwise comparisons of the probability that moose would leave a site after exposure to an acoustic stimulus, using Tukey's honest significant difference tests.

Pairwise comparison	Estimate	Standard error	*p* Value
Dog – Silent	*2.81*	*0.39*	*<.001*
Human – Silent	*4.75*	*0.42*	*<.001*
Bird – Silent	*2.05*	*0.40*	*<0.001*
Human – Dog	*1.94*	*0.37*	*<.001*
Bird – Dog	−0.76	0.36	.146
Bird – Human	*−2.71*	*0.38*	*<.001*

*Note*: Comparisons with a significant effect (*α* = .05) are italicized.

### 3.2 Behaviors—Vigilant, feeding, time out of frame

**FIGURE 3:**
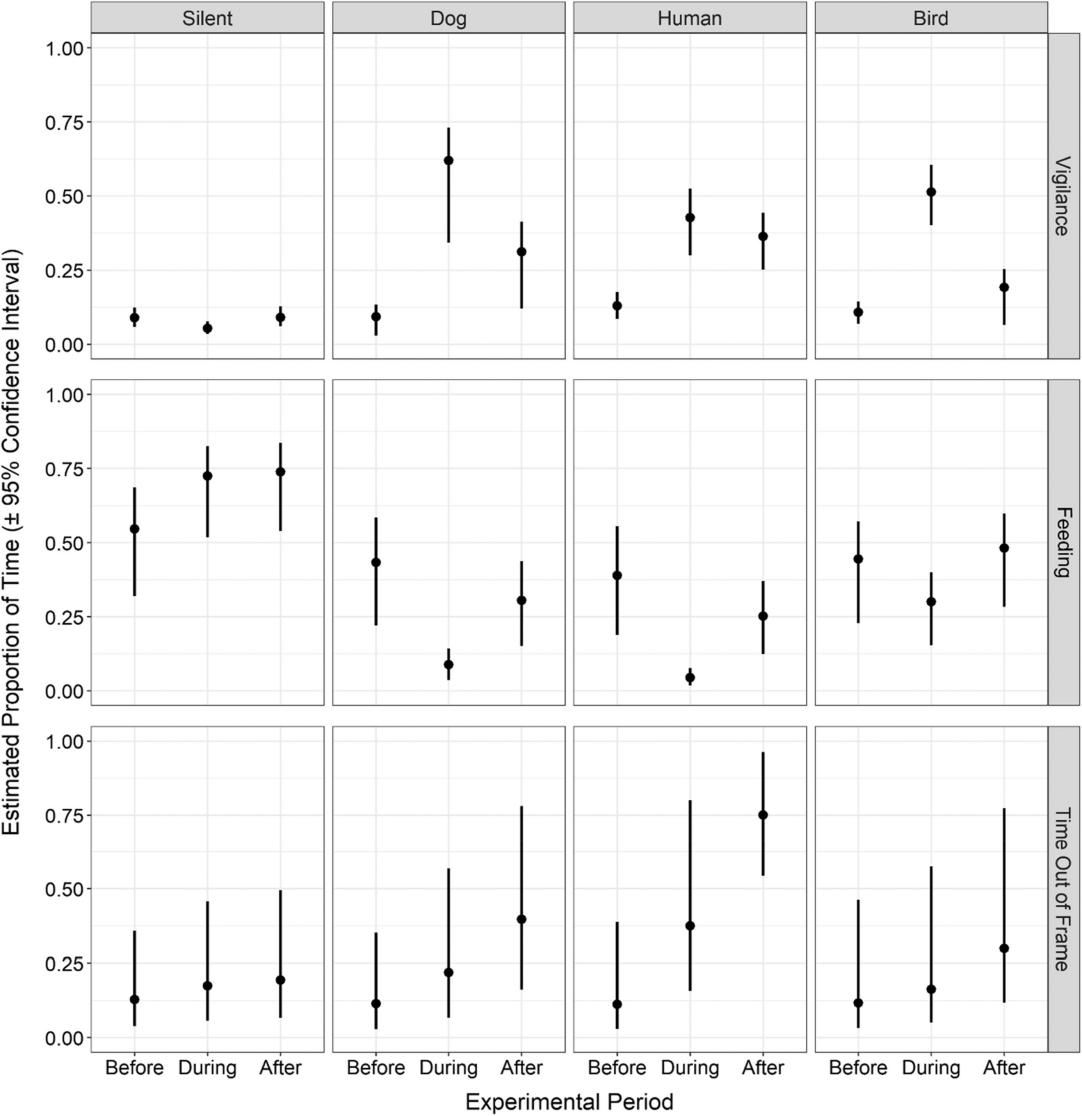
The estimated proportion of time moose spent vigilant (top), feeding (middle), or out of frame (bottom) before, during, and after they were exposed to either a silent control or dog, human, or bird acoustic stimuli. These estimations show the changes in the proportion of time with the experimental period, while holding all other variables constant.

In all three models, all explanatory variables from the global model were present in top models (∆AICc < 2; Table 7), and therefore included in the conditionally averaged logistic regression (Table 8). Before exposure to acoustic stimuli, moose spent, on average 2 s vigilant, 9 s feeding, and 2 s out of the frame. When exposed to any acoustic stimuli, moose spent significantly more time vigilant (average: 8 s, *p* < .001; Tables 8 and 9; Figure 3) and significantly less time feeding (average: 3 s, *p* < .001), compared with the silent control. Moose spent the most time vigilant when exposed to dog stimulus (average: 10 s, *p* < .001; Tables 8 and 9) while spending equal amounts of time vigilant when exposed to the human stimulus or bird stimulus (*p* = .521; Table 9). Moose spent significantly less time feeding when exposed to dog stimulus or human stimulus than the bird stimulus (average dog: 2 s, average human: 1 s, *p* = .01; Table 9), or silent control (*p* < .001; Table 9). Finally, compared with the silent control events, moose spent significantly more time out of the frame when exposed to the human stimulus (average: 8 s, *p* = .001; Table 9).

**TABLE 7:** AICc table for the candidate models describing the proportion of time moose spent vigilant, feeding, or out of frame before, during, and after being exposed to an acoustic stimulus.

Candidate models	AICc	∆AICc	AICc weight
Vigilance			
Intercept + Period + Stimulus + Period × Stimulus + Trial day + Age	1581.8	0	0.234
Intercept + Period + Stimulus + Period × Stimulus + Trial day + Age + Wind	1582.7	0.84	0.154
Intercept + Period + Stimulus + Period × Stimulus + Trial day + Age + Sex	1583.4	1.53	0.109
Intercept + Period + Stimulus + Period × Stimulus + Trial day + Age + Rain	1583.6	1.74	0.098
Intercept + Period + Stimulus + Period × Stimulus + Trial day + Age + Sex + Wind	1584.1	2.22	0.077
Null model	1908	326.2	0
Feeding			
Intercept + Period + Stimulus + Period × Stimulus + Trial day + Age + Sex + Rain	2228.9	0	0.298
Intercept + Period + Stimulus + Period × Stimulus + Trial day + Age + Sex + Rain + Wind	2229.4	0.52	0.23
Intercept + Period + Stimulus + Period × Stimulus + Trial day + Sex + Rain	2229.8	0.92	0.188
Intercept + Period + Stimulus + Period × Stimulus + Trial day + Sex + Rain + Wind	2230.3	1.46	0.144
Intercept + Period + Stimulus + Period × Stimulus + Trial day + Age + Sex	2232.2	3.36	0.056
Null model	2639	410.14	0
Time out of frame			
Intercept + Period + Stimulus + Period × Stimulus + Trial day + Age + Sex + Rain + Wind	1993	0	0.454
Intercept + Period + Stimulus + Period × Stimulus + Trial day + Age + Sex + Wind	1993.7	0.68	0.323
Intercept + Period + Stimulus + Period × Stimulus + Trial day + Age + Sex + Rain	1995.8	2.79	0.113
Null model	2429.7	436.71	0

*Note*: We display the top‐performing models (∆AICc < 2), the first model ∆AICc > 2, and the null model for each behavior.

**TABLE 8:** Conditionally averaged model output of the top‐performing models (∆AICc < 2; Table 6) for the proportion of time moose spent vigilant, feeding, or out of frame before, during, and after being exposed to an acoustic stimulus.

	Vigilance			Feeding			Time out of frame		
Coefficient	Estimate	Standard error	*p* Value	Estimate	Standard error	*p* Value	Estimate	Standard error	*p* Value
Intercept	*−2.07*	*0.30*	*<.001*	−0.22	0.23	.334	*−1.37*	*0.37*	*<.001*
During	−0.52	0.35	.142	*0.76*	*0.19*	*<.001*	0.41	0.24	.090
After	0.04	0.31	.895	*0.85*	*0.19*	*<.001*	*0.57*	*0.24*	*.018*
Dog stimulus	0.03	0.35	.931	−0.40	0.21	.053	−0.31	0.32	.334
Human stimulus	0.39	0.32	.233	*−0.55*	*0.21*	*.009*	−0.37	0.33	.260
Bird stimulus	0.15	0.35	.661	−0.33	0.21	.121	−0.33	0.33	.312
During × Dog stimulus	*3.39*	*0.49*	*<.001*	*−2.91*	*0.39*	*<.001*	0.48	0.41	.247
After × Dog stimulus	*1.48*	*0.47*	*.002*	*−1.49*	*0.33*	*<.001*	*1.32*	*0.40*	*.001*
During × Human stimulus	*2.16*	*0.47*	*<.001*	*−3.48*	*0.49*	*<.001*	*1.43*	*0.41*	*<.001*
After × Human stimulus	*1.21*	*0.50*	*.016*	*−1.64*	*0.43*	*<.001*	*3.13*	*0.42*	*<.001*
During × Bird stimulus	*2.73*	*0.48*	*<.001*	*−1.46*	*0.32*	*<.001*	0.04	0.44	.930
After × Bird stimulus	0.66	0.48	.169	*−0.74*	*0.32*	*.020*	0.81	0.41	.050
Trial day	*−0.01*	*0.01*	*.026*	*0.02*	*0.00*	*<.001*	*−0.02*	*0.01*	*<.001*
Juvenile	*−1.21*	*0.40*	*.003*	0.54	0.32	.085	*−1.00*	*0.27*	*<.001*
Female with calf	−0.23	0.37	.536	−0.38	0.28	.176	*0.58*	*0.29*	*.043*
Male	0.26	0.15	.073	0.02	0.13	.864	*−0.34*	*0.15*	*.022*
Adult (unknown sex)	0.01	0.26	.958	*−1.01*	*0.24*	*<.001*	*1.76*	*0.18*	*<.001*
Rain	0.04	0.07	.586	*−0.18*	*0.08*	*.025*	0.12	0.07	.097
Wind	−0.07	0.06	.279	0.06	0.05	.220	*−0.12*	*0.06*	*.035*

*Note*: The intercepts are the responses of adult females (without calves) in the before the period of silent events on day 0 of the experiments, with no rain and wind. Variables with a significant effect (*α* = .05) are italicized.

**TABLE 9:** Pairwise comparisons of the proportion of time moose spent vigilant, feeding, or out of frame among each acoustic stimulus, using Tukey's honest significant difference tests.

Pairwise comparison	Vigilance			Feeding			Time out of frame		
	Estimate	Standard error	*p* Value	Estimate	Standard error	*p* Value	Estimate	Standard error	*p* Value
Overall									
Dog – Silent	0.04	0.35	1.000	−0.40	0.21	.201	−0.30	0.32	.776
Human – Silent	0.38	0.33	.653	−0.55	0.21	.045	−0.38	0.33	.654
Bird – Silent	0.16	0.35	.970	−0.32	0.21	.418	−0.33	0.33	.741
Human – Dog	0.34	0.38	.802	−0.14	0.24	.930	−0.07	0.38	.997
Bird – Dog	0.12	0.39	.991	0.08	0.24	.986	−0.03	0.38	1.000
Bird – Human	−0.22	0.37	.932	0.23	0.24	.787	0.04	0.38	.999
Before									
Dog – Silent	0.00	0.36	1.000	−0.47	0.20	.101	−0.27	0.34	.855
Human – Silent	0.35	0.33	.719	−0.60	0.21	.019	−0.45	0.36	.581
Bird – Silent	0.10	0.35	.991	−0.37	0.21	.288	−0.39	0.36	.696
Human – Dog	0.34	0.38	.803	−0.14	0.24	.936	−0.18	0.41	.971
Bird – Dog	0.10	0.40	.994	0.09	0.24	.978	−0.12	0.41	.991
Bird – Human	−0.24	0.37	.915	0.23	0.24	.763	0.06	0.42	.999
During									
Dog – Silent	*3.55*	*0.35*	*<.001*	*−3.35*	*0.34*	*<.001*	0.19	0.27	.886
Human – Silent	*2.62*	*0.34*	*<.001*	*−4.07*	*0.45*	*<.001*	*1.05*	*0.25*	*<.001*
Bird – Silent	*2.97*	*0.35*	*<.001*	*−1.78*	*0.25*	*<.001*	−0.29	0.30	.749
Human – Dog	*−0.93*	*0.26*	*.002*	−0.72	0.52	.496	*0.85*	*0.28*	*.012*
Bird – Dog	−0.58	0.27	.123	*1.57*	*0.37*	*<.001*	−0.49	0.32	.420
Bird – Human	0.35	0.26	.531	*2.29*	*0.47*	*<.001*	*−1.34*	*0.31*	*<.001*
After									
Dog – Silent	*1.57*	*0.31*	*<.001*	*−1.90*	*0.27*	*<.001*	*0.99*	*0.23*	*<.001*
Human – Silent	*1.73*	*0.39*	*<.001*	*−2.27*	*0.38*	*<.001*	*2.69*	*0.26*	*<.001*
Bird – Silent	*0.84*	*0.34*	*.060*	*−1.03*	*0.24*	*<.001*	0.46	0.25	.240
Human – Dog	0.16	0.38	.975	−0.37	0.41	.792	*1.70*	*0.27*	*<.001*
Bird – Dog	−0.73	0.32	.103	*0.87*	*0.29*	*.014*	−0.52	0.26	.187
Bird – Human	−0.89	0.40	.110	*1.24*	*0.40*	*.010*	*−2.22*	*0.28*	*<.001*

*Note*: Pairwise tests were performed for the data overall, and also for each period of the trial, before, during, and after. Comparisons with a significant effect (*α* = 0.05) are italicized.

After exposure to acoustic stimuli, moose had variable responses (Figure 3). After exposure to the dog stimulus, moose continued to spend significantly more time vigilant (*p* = .002) and out of the frame (*p* = .001), and less time feeding (*p* < .001), than before the stimulus. After exposure to the human stimulus, moose spent more time vigilant (*p* = .016) or out of the frame (*p* < .001), and less time feeding (*p* < .001) than before exposure to the stimulus. Finally, after exposure to the bird stimulus, moose returned to the same amount of time spent vigilant (*p* = .169), and out of frame (*p* = .05), however, they spent less time feeding (*p* = .020) compared with before exposure to the bird stimulus.

Overall, juveniles spend less time vigilant (*p* = .003), and out of the frame (*p* < .001) but similar time feeding (*p* = .085) as adults. Females with calves spent more time out of the frame than females without calves (*p* < .043); while males were overall more vigilant and spent more time out of frame than adult females without calves (*p* = .073 and *p* = .022, respectively).

Moose spent significantly less time feeding when rain increased (*p* = .025; Table 7), and less time out of the frame when wind increased (*p* = .035). The other behaviors were not significantly affected by wind or rain. Moose spent significantly less time vigilant (*p* = .026), significantly more time feeding (*p* < .001), and less time out of frame (*p* < .001) as the experiment progressed over the 50 days of data collection (Table 7).

### 3.3 Time between events at the same site

In the time between events model, all explanatory variables from the global model were included in the top models (∆AICc < 2; Table 10), and therefore included in the conditionally averaged Gamma regression (Table 11).

Moose took a longer time between events after exposure to any of the acoustic stimuli, compared to after a silent control (Tables 11 and 12). The longest time between events was after a human stimulus, but there was no significant difference among the time bewteen events after exposure to either of the stimuli (Tables 11 and 12). The time between events was not significantly affected by wind, rain, or trial day (Table 11).

**TABLE 10:** AICc table for the candidate models describing the time between events at a site after being exposed to an acoustic stimulus.

Candidate model	AICc	∆AICc	AICc weight
Intercept + Stimulus before	3850.8	0	0.314
Intercept + Stimulus before + Trial day	3852	1.26	0.167
Intercept + Stimulus before + Rain	3852.1	1.33	0.161
Intercept + Stimulus before + Wind	3852.7	1.91	0.12
Intercept + Stimulus before + Trial day + Rain	3853.4	2.64	0.084
Null model	3907.6	56.83	0

*Note*: We display the top‐performing models (∆AICc < 2), the first model ∆AICc > 2, and the null model.

**TABLE 11:** Conditionally averaged model output for the amount of time between events at a site after being exposed to an acoustic stimulus.

Coefficient	Estimate	Standard error	*p* Value
Intercept	*0.186*	*0.023*	*<.0001*
Previous stimulus: Dog	*−0.104*	*0.026*	*<.001*
Previous stimulus: Human	*−0.131*	*0.024*	*<.001*
Previous stimulus: Bird	*−0.090*	*0.028*	*.002*
Trial day	0.000	0.001	.494
Rain	−0.004	0.005	.430
Wind	0.002	0.007	.796

*Note*: The intercept is an event after a silent stimulus, on events on day 0 of the experiments, with no rain and wind. Variables with a significant effect (*α* = .05) are italicized.

**TABLE 12:** Pairwise comparisons of the amount of time between events at a site after being exposed to an acoustic stimulus, using Tukey's honest significant difference tests.

Pairwise comparison	Estimate	Standard error	*p* Value
Dog – Silent	*−0.10*	*0.03*	*<.0001*
Human – Silent	*−0.13*	*0.02*	*<.001*
Bird – Silent	*−0.09*	*0.03*	*.008*
Human – Dog	−0.03	0.02	.454
Bird – Dog	0.01	0.02	.946
Bird – Human	0.04	0.02	.234

*Note*: Comparisons with a significant effect (*α* = .05) are italicized.

## 4 DISCUSSION

Acoustic stimuli were effective at inducing consistent behavioral reactions in moose. Of the four stimuli used in this experiment, moose displayed the strongest anti‐predatory reactions when exposed to the human voice and barking dog. Moose were more likely to flee and take longer to visit a site when exposed to the human voice (Tables 5 and 11). While exposed to the dog stimulus, moose were more vigilant and fed less, despite remaining at the site more than when exposed to the human stimulus (Table 8, Figure 3). These reactions match what one would expect in reality, and also corroborate other studies (e.g., Crawford et al., 2022; Widén et al., 2022). Given the degree of threat, exposure to humans may warrant the extra energy expenditure to flee a site (Proffitt et al., 2009; Zbyryt et al., 2018). Contrastingly, the increased alertness in response to dog barking is how moose react to dogs during the hunt—become observant of the dog and stand still while locating the dog (Svenska Jägareförbundet, 2012).

Contrary to our expectations, moose were more responsive towards the bird stimulus than they were to the silent controls. Moose exposed to the bird stimulus spent less time feeding and more time vigilant compared with the silent controls (Table 8, Figure 3). After exposure to the bird stimulus, moose often returned to the same behavior as before exposure (Figure 3). Moose were unlikely to flee from the bird stimulus (Table 5), and their reactiveness quickly reduced over the duration of the experiment (Figure 2). The combination of these results suggests that the response detected in moose could be attributed to the suddenness in the appearance of acoustic stimuli rather than towards the information conveyed by the stimuli (Brown et al., 2013, 2015).

Exposure duration and frequency influence the extent to which an animal habituates to a stimulus (Biedenweg et al., 2011; Blumstein, 2016; Bomford & O'Brien, 1990; Winslow et al., 2002). In our experimental setup, we ran the risk of exposing the same individuals to the stimuli intensively over a short period of time,  as the same individuals likely visited the same saltlick stones repeatedly, even though each saltlick stone was an independent site. It is also likely that we underestimated the amount of times an individual was exposed to an acoustic stimulus, since individuals that were not captured on video but were in the vicinity of the saltlick stone, would have also heard the stimulus. This intensity of exposure likely contributed to the desensitization we found over the duration of the experiment (Figure 2; Babińska‐Werka et al., 2015). At the Grimsö Wildlife Research Area, we do not have marked moose individuals, therefore, it is not possible to say with certainty how much the same individuals were exposed to the acoustic stimuli. To deduce the true desensitization or habituation effects, it would be interesting to conduct a similar study on marked individuals.

Females, with or without calves, and juveniles displayed the strongest anti‐predatory responses (Tables 5 and 8). At the start of the experiments, juveniles (i.e., 1–2 years old) accompanied females to the saltlick stone sites, and as the experiments progressed, females gave birth to calves and returned to the sites with new calves. It is not surprising that juveniles and females responded similarly to one another. Since juveniles, particularly calves, are at the highest risk of predation, females reacting strongly could be a form of protection of the young (Johnsen, 2013). Adult males, on the other hand, were more likely to remain at the sites and less likely to flee as a result of the acoustic stimuli. Although males spent more time out of the frame than females, they were not fleeing or running away from the site in panic and thus may have just been out of frame and out of view but still close to the saltlick stone. Adult males may have been less reactive to the acoustic stimuli since these experiments were conducted outside of the hunting season, and the expenditure of energy towards avoiding the sites was not warranted if the threat was not true.

The results of this study are highly dependent upon the context in which the experiments were conducted. First, the choice of attractant may result in a different trade‐off in response. While moose are attracted to saltlick stones, a more desirable attractant, such as young coniferous plantations (Äbin; Kalén et al., 2018; Kjellander, 2007), may lead to different results. Secondly, exposure to humans could have influenced how tolerant the moose in this experiment were towards the acoustic stimuli. Moose in the Scandinavian forests are highly reactive to human presence, and often change their spatial patterns to avoid humans, despite the amount of exposure to humans (Neumann, 2009). This is supported by our study, as over the course of the experiment, moose were less and less likely to flee when exposed to the dog and bird stimuli while maintaining the strongest reaction to the human stimulus (Figure 2). In environments with less human exposure, the results may turn out to be different. This could also be influenced by the amount of cover available for individuals to retreat to. Thirdly, behaviors can be plastic and can change throughout the year. For example, during hunting seasons, ungulates tend to be more reactive than outside the hunting season (Stankowich & Blumstein, 2005). This experiment was conducted outside of the hunting season, which may have contributed to the desensitization of moose towards the dog stimulus. Similarly, in this experiment, the acoustic stimuli were not followed by a true threat. This lack of danger may not warrant energy expenditure by the moose and after habitual exposure, they may become less reactive in order to conserve energy (Babińska‐Werka et al., 2015). The results we derived from this experiment may be different in a different habitat context or at another time of the year.

The type of stimuli used has a strong influence on the response one can elicit. In this experiment, we presumed that the moose would perceive the threatening and nonthreatening stimuli as we heard them; however, the reactiveness towards the nonthreatening bird stimuli suggests that this may not have been entirely the case. It is important to imitate stimuli as closely as possible to natural sounds, in pitch, frequency, and volume, to reduce the novelty of the stimuli and any responses that may result as a consequence. To fully test the theory that moose are reacting to the information conveyed by the acoustic stimulus, it would be interesting to explore how moose behave when exposed to stimuli that do not carry any information, such as an artificial electronic sound, a bell, siren, or similar. In this study, we used a single exemplar to demonstrate the responsiveness of moose to specific stimuli. As such, we cannot discuss the generalized response of moose to a class of sounds (i.e., all dog sounds; all human sounds); however, we can form conclusions based on the response of moose to the particular playbacks used in this experiment. Furthermore, it is not possible to know whether animals do hear and perceive the sound as we presume they do, or that there are no external influences such as electronic noise from the system that influence the response of individuals towards the playbacks. Thus, future studies would benefit from using richer repertoires with more exemplars in order to draw conclusions with the class of sounds while minimizing the effects of external influences (Kroodsma et al., 2001). This could help clarify if the reaction is true to the information in the acoustic stimuli or if it is a response to the particular sound files used.

Evoking flight responses using acoustic stimuli may be useful to manage human‐wildlife conflicts, where the desired action is to reduce visitation by wildlife. This could be useful to reduce the occurrence of wildlife, for example, in agricultural areas, forest plantations, or on roads and railways (e.g., Babińska‐Werka et al., 2015; Gilsdorf et al., 2004; Hildreth et al., 2013; Honda, 2019; Shimura et al., 2018; Widén et al., 2022). Like our findings, other studies suggest that human voices are a strong cue to elicit flight and avoidance in wildlife (e.g., Clinchy et al., 2016; Crawford et al., 2022; Epperly et al., 2021; Smith et al., 2017; Suraci et al., 2019; Widén et al., 2022). In order to use this response successfully in a management scenario, one must strive to avoid habituation of the target to the stimuli (Blumstein, 2016), which may be achieved through controlling the use and display of the stimuli to reduce repetition (Babińska‐Werka et al., 2015; Blumstein, 2016), or by using the stimuli as a warning that is followed by a real threat, such as an approaching train (Babińska‐Werka et al., 2015; Seiler et al., 2017; Shimura et al., 2018). In‐situ tests are essential to determine the right stimuli to use, and what frequency of recurrence is effective. Furthermore, as previously discussed, the context of the landscape can influence the response in wildlife (Epperly et al., 2021; Stankowich & Blumstein, 2005), so careful consideration of predation pressure, human pressure, and the availability of cover is essential as they may influence how successful acoustic stimuli are in management situations. While the approach is promising in eliciting consistent behavior over time, tests and further context‐specific studies are needed to confirm that.

In this experiment, we were able to demonstrate that acoustic stimuli can be used to induce innate, anti‐predatory behaviors in moose. Acoustic stimuli, particularly those associated with human presence, may be an effective method at eliciting consistent anti‐predatory behavior in ungulates and are likely a reliable tool to use in management, for example by inducing flight from sites of conflict. Further studies into the validity of this theory are warranted and deserve attention as methods to reduce human‐wildlife conflicts are developed.

## AUTHOR CONTRIBUTIONS

**Manisha Bhardwaj:** Conceptualization (equal); data curation (lead); formal analysis (lead); funding acquisition (equal); investigation (lead); methodology (equal); project administration (equal); supervision (lead); validation (equal); visualization (equal); writing – original draft (lead); writing – review and editing (lead). **Denice Lodnert:** Data curation (supporting); methodology (lead); visualization (lead); writing – original draft (supporting); writing – review and editing (supporting). **Mattias Olsson:** Conceptualization (equal); data curation (equal); funding acquisition (equal); investigation (equal); methodology (equal); supervision (equal); writing – review and editing (equal). **Aina Winsvold:** Conceptualization (equal); funding acquisition (equal); project administration (equal); writing – review and editing (equal). **Svein Morten Eilertsen:** Conceptualization (equal); funding acquisition (equal); project administration (equal); writing – review and editing (equal). **Petter Kjellander**: Formal analysis (supporting); funding acquisition (supporting); methodology (equal); writing – review and editing (equal). **Andreas Seiler:** Conceptualization (equal); data curation (equal); funding acquisition (equal); project administration (equal); resources (equal); writing – review and editing (equal).

## Supporting information


Appendix S1

## Data Availability

Data is available at https://doi.org/10.6084/m9.figshare.17128679 has been reserved and will be published upon acceptance.
