# Supplementary material for: Inducing fear using acoustic stimuli—A behavioral experiment on moose (Alces alces) in Sweden
Source: Ecol Evol. 2022 Nov 16;12(11):e9492. doi: 10.1002/ece3.9492 (PMC9667297; doi:10.1002/ece3.9492)
Supplement: Supplementary file 1 — Appendix S1 [file ECE3-12-e9492-s001.docx]

# Supplementary Information

Supplementary Information Table 1. Average time in seconds ± standard deviation for the different behaviours before, during and after exposure to the acoustic stimuli.

|  | ***Before*** | ***During*** | ***After*** |
| --- | --- | --- | --- |
| ***Feeding*** |  |  |  |
| Dog | 8.37 ± 8.56 | 2.41 ± 4.37 | 4.47 ± 7.31 |
| Human | 7.87 ± 8.42 | 1.40 ± 2.77 | 1.59 ± 4.95 |
| Bird | 8.72 ± 8.53 | 5.28 ± 7.36 | 7.55 ± 8.74 |
| Silent | 10.33 ± 8.91 | 12.05 ± 8.87 | 11.90 ± 8.95 |
| ***Fleeing*** |  |  |  |
| Dog | 0.06 ± 0.53 | 1.78 ± 3.18 | 0.45 ± 1.53 |
| Human | 0.06 ± 0.37 | 3.31 ± 3.35 | 0.44 ± 1.74 |
| Bird | 0.16 ± 0.87 | 0.50 ± 1.69 | 0.22 ± 1.09 |
| Silent | 0.11 ± 0.75 | 0.04 ± 0.45 | 0.01 ± 0.23 |
| ***Social interaction*** | |  |  |
| Dog | 0.00 ± 0.00 | 0.01 ± 0.16 | 0.00 ± 0.00 |
| Human | 0.23 ± 2.06 | 0.02 ± 0.24 | 0.00 ± 0.00 |
| Bird | 0.20 ± 1.42 | 0.02 ± 0.18 | 0.00 ± 0.00 |
| Silent | 0.00 ± 0.00 | 0.02 ± 0.21 | 0.02 ± 0.26 |
| Standing |  |  |  |
| Dog | 3.25 ± 5.37 | 0.65 ± 2.39 | 0.83 ± 3.05 |
| Human | 3.10 ± 5.51 | 0.49 ± 1.75 | 0.21 ± 1.47 |
| Bird | 2.74 ± 4.85 | 1.01 ± 3.41 | 0.81 ± 2.91 |
| Silent | 2.78 ± 5.71 | 2.21 ± 5.27 | 1.46 ± 4.28 |
| ***Time out of frame*** | |  |  |
| Dog | 2.03 ± 4.93 | 4.56 ± 7.51 | 7.97 ± 9.24 |
| Human | 2.06 ± 4.85 | 7.70 ± 6.91 | 15.07 ± 8.37 |
| Bird | 2.22 ± 5.14 | 3.46 ± 7.31 | 5.87 ± 8.53 |
| Silent | 2.36 ± 5.42 | 3.55 ± 7.34 | 4.07 ± 7.81 |
| ***Vigilant*** |  |  |  |
| Dog | 2.14 ± 4.97 | 9.93 ± 7.35 | 4.65 ± 6.82 |
| Human | 2.68 ± 5.46 | 6.55 ± 6.61 | 2.28 ± 6.62 |
| Bird | 2.07 ± 5.27 | 8.18 ± 7.72 | 3.41 ± 5.82 |
| Silent | 1.81 ± 4.74 | 1.17 ± 3.46 | 1.51 ± 4.35 |
| ***Walking*** |  |  |  |
| Dog | 4.16 ± 4.95 | 0.65 ± 1.89 | 1.63 ± 3.82 |
| Human | 4.00 ± 5.04 | 0.53 ± 1.97 | 0.42 ± 1.87 |
| Bird | 3.88 ± 5.22 | 1.55 ± 3.25 | 2.14 ± 4.20 |
| Silent | 2.61 ± 4.29 | 0.96 ± 2.73 | 1.02 ± 3.19 |

Supplementary Information Table 2. Number of activations per stimuli per site. The system could have been activated by a wildlife other than moose, so the total number of activations as well as those activations by moose are presented. Moose within the vicinity of the saltlick-stone likely heard the stimuli, even if they were not the ones to activate the system.

| Site | ***Stimuli*** | ***Total Number of Activations*** | ***Activations by Moose*** |
| --- | --- | --- | --- |
| 1 | Dog | 50 | 28 |
|  | Human | 37 | 27 |
|  | Bird | 44 | 22 |
|  | Silent | 101 | 38 |
| 2 | Dog | 86 | 18 |
|  | Human | 88 | 17 |
|  | Bird | 76 | 15 |
|  | Silent | 511 | 52 |
| 3 | Dog | 28 | 12 |
|  | Human | 38 | 18 |
|  | Bird | 38 | 14 |
|  | Silent | 97 | 36 |
| 4 | Dog | 99 | 29 |
|  | Human | 109 | 26 |
|  | Bird | 104 | 29 |
|  | Silent | 877 | 72 |
| 5 | Dog | 71 | 7 |
|  | Human | 59 | 6 |
|  | Bird | 57 | 2 |
|  | Silent | 579 | 6 |
| 6 | Dog | 75 | 20 |
|  | Human | 73 | 20 |
|  | Bird | 86 | 27 |
|  | Silent | 360 | 31 |
| 7 | Dog | 19 | 5 |
|  | Human | 23 | 3 |
|  | Bird | 31 | 7 |
|  | Silent | 155 | 12 |
| 8 | Dog | 59 | 25 |
|  | Human | 66 | 27 |
|  | Bird | 56 | 18 |
|  | Silent | 156 | 32 |


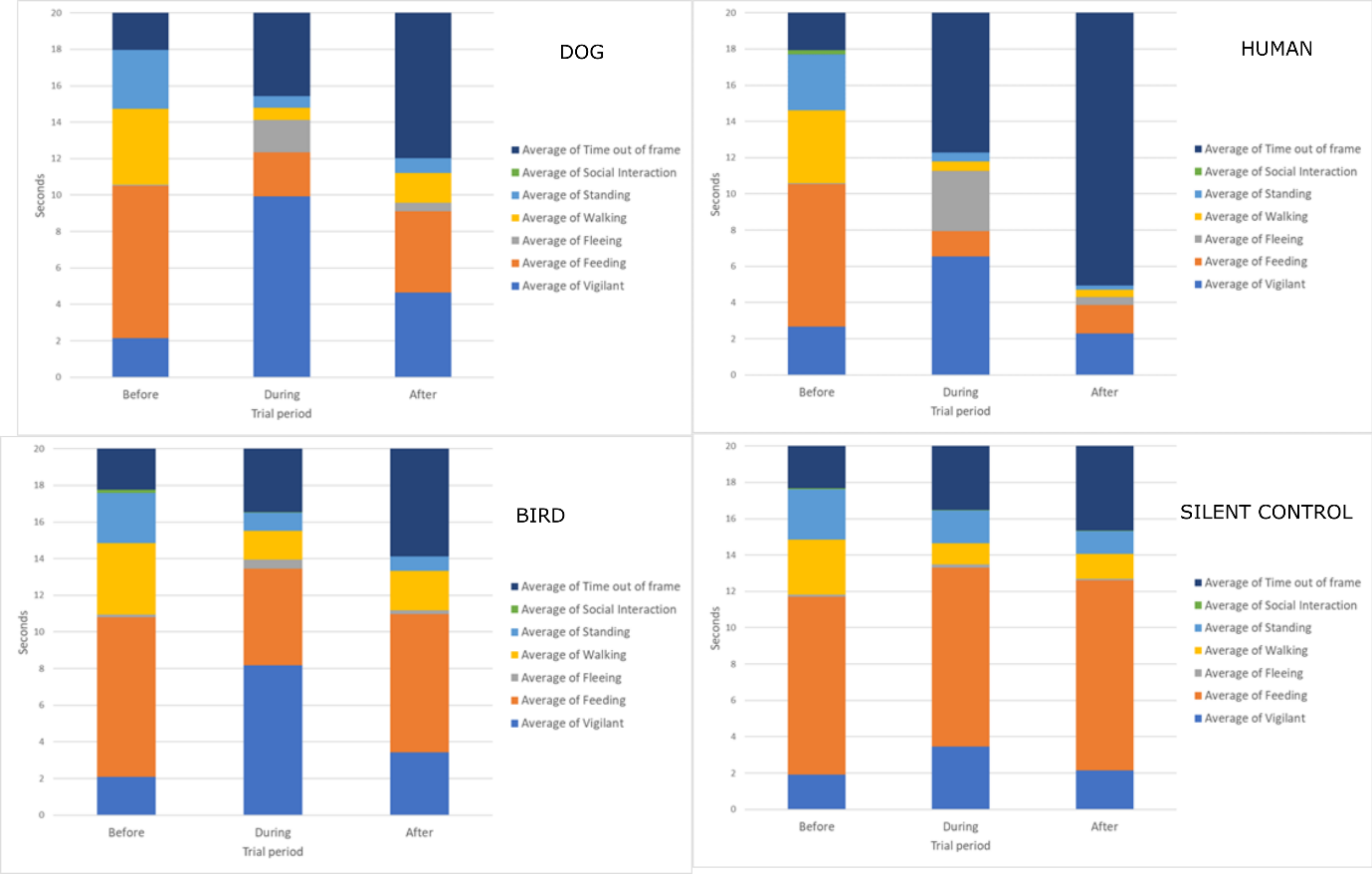


Supplementary Information Figure 1. Average proportions of time in seconds that moose spent displaying the different behaviours in the ethogram before, during and after being exposed to dog (top right), human (top left), bird (bottom right) and silent (bottom left) stimuli.
